# Supplementary material for: Fine-Tuning Tomato Agronomic Properties by Computational Genome Redesign
Source: PLoS Comput Biol. 2012 Jun 7;8(6):e1002528. doi: 10.1371/journal.pcbi.1002528 (PMC3369923; doi:10.1371/journal.pcbi.1002528)
Supplement: Figure S1 — Synthetic biology of tomato fruit vs computer science. (PDF) [file pcbi.1002528.s005.pdf]

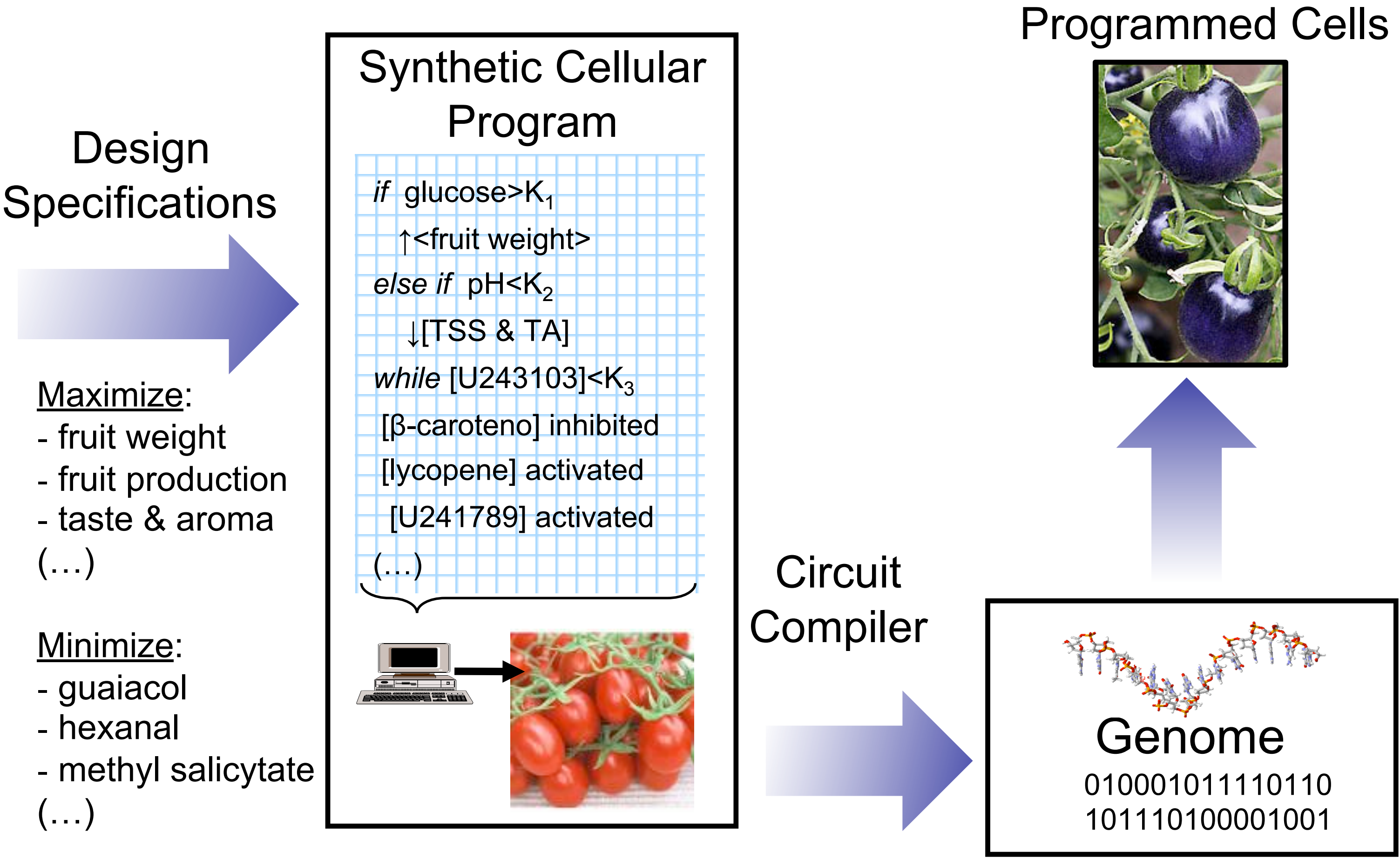

Design Specifications
Maximize:
- fruit weight
- fruit production
- taste & aroma
(…)
Minimize:
- guaiacol
- hexanal
- methyl salicytate
(…)
Synthetic Cellular Program
if glucose>K_1
↑<fruit weight>
else if pH<K_2
↓[TSS & TA]
while [U243103]<K_3
[β-caroteno] inhibited
[lycopene] activated
[U241789] activated
(…)
Circuit Compiler
Genome
010001011110110
101110100001001
Programmed Cells
