# Supplementary material for: Fine-Tuning Tomato Agronomic Properties by Computational Genome Redesign
Source: PLoS Comput Biol. 2012 Jun 7;8(6):e1002528. doi: 10.1371/journal.pcbi.1002528 (PMC3369923; doi:10.1371/journal.pcbi.1002528)
Supplement: Figure S2 — From data to global models to redesign using an approach based on synthetic biology. (PDF) [file pcbi.1002528.s006.pdf]

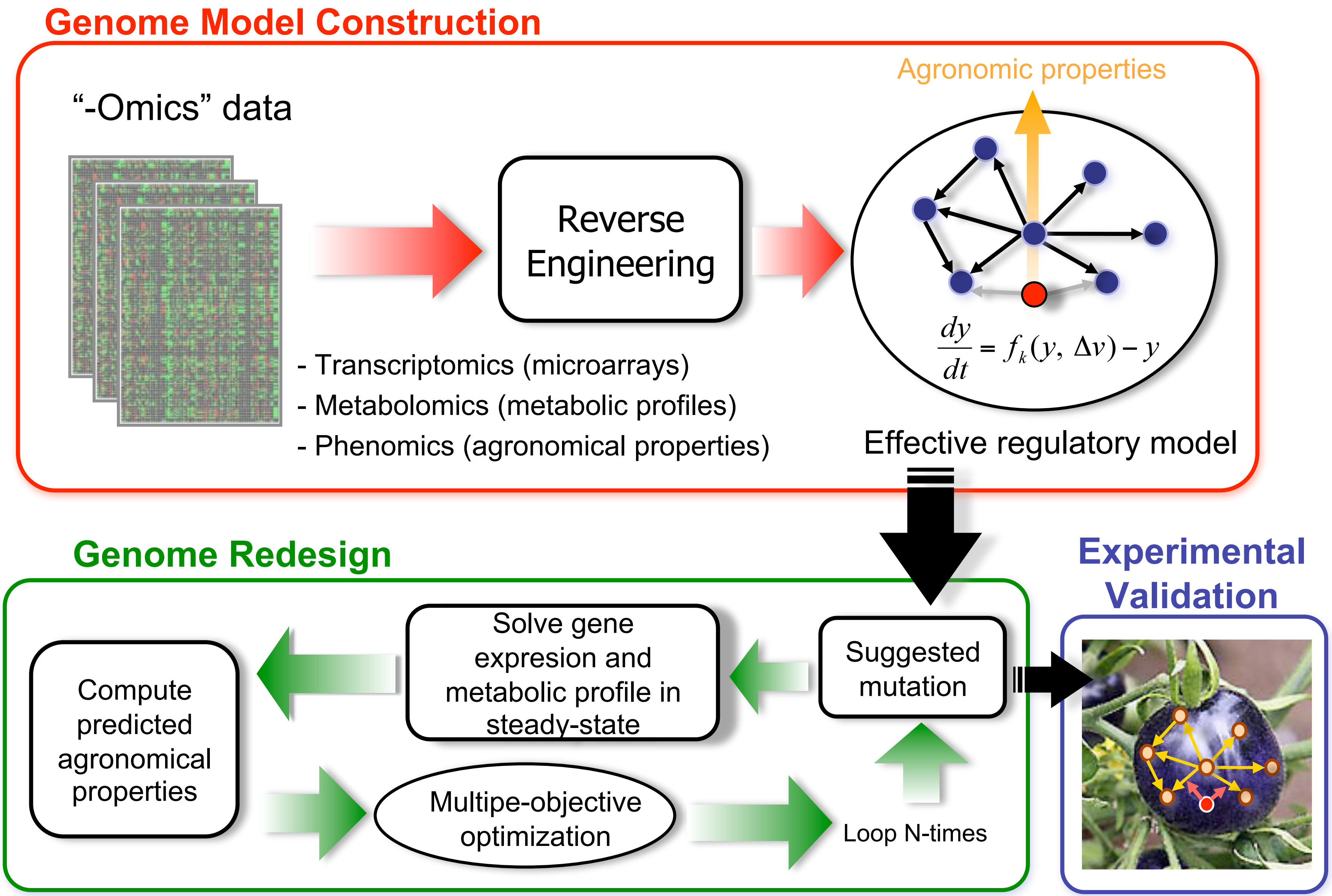

Genome Model Construction
"-Omics" data
Reverse Engineering
Agronomic properties
$\frac{dy}{dt} = f_k(y, \Delta v) - y$
- Transcriptomics (microarrays)
- Metabolomics (metabolic profiles)
- Phenomics (agronomical properties)
Effective regulatory model
Genome Redesign
Experimental Validation
Compute predicted agronomical properties
Solve gene expresion and metabolic profile in steady-state
Suggested mutation
Multipe-objective optimization
Loop N-times
