# Supplementary figures and images for: Fine-Tuning Tomato Agronomic Properties by Computational Genome Redesign
Source: PLoS Comput Biol. 2012 Jun 7;8(6):e1002528. doi: 10.1371/journal.pcbi.1002528 (PMC3369923; doi:10.1371/journal.pcbi.1002528)

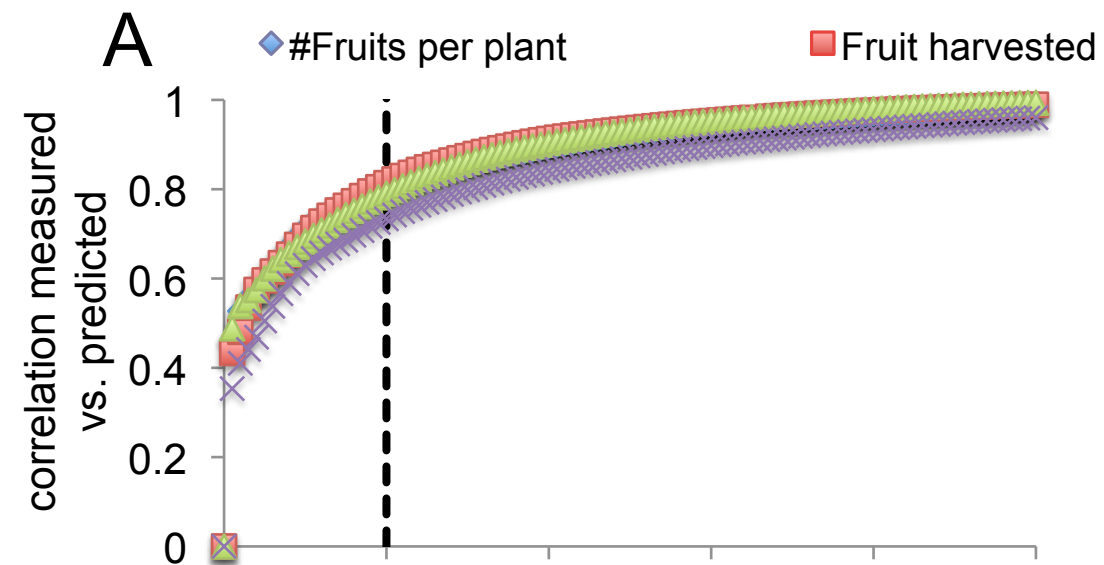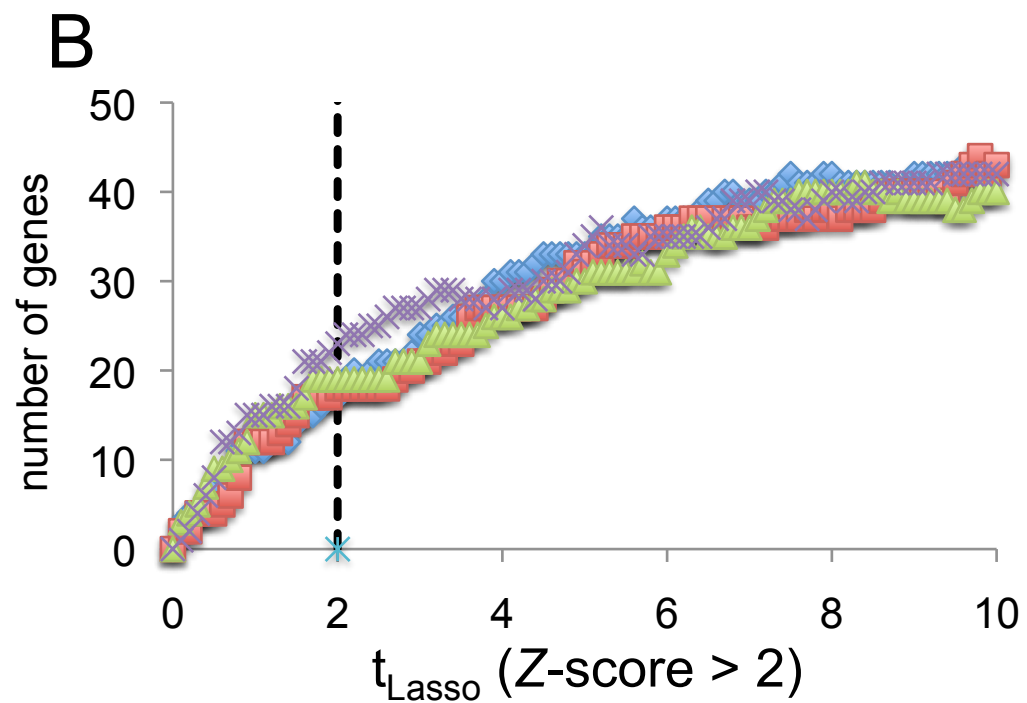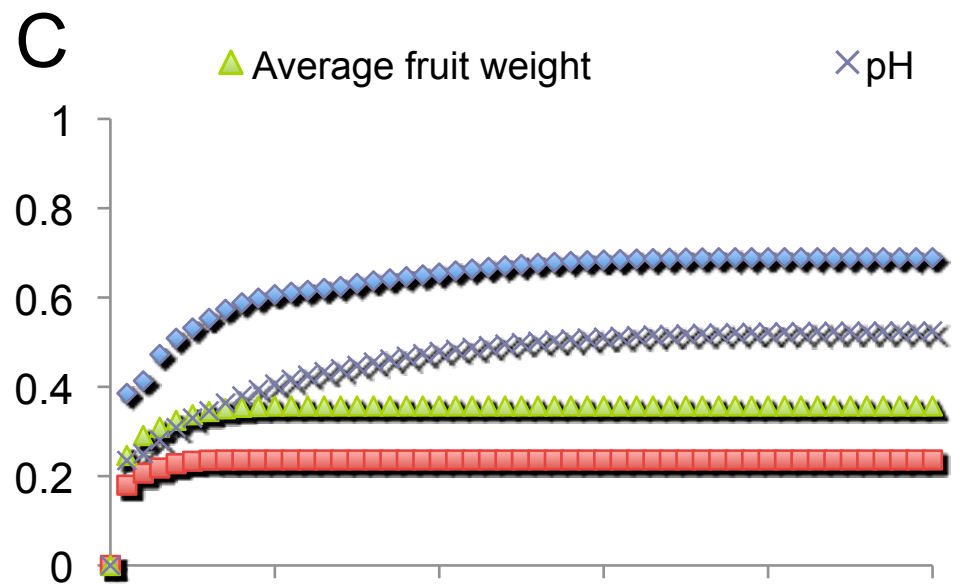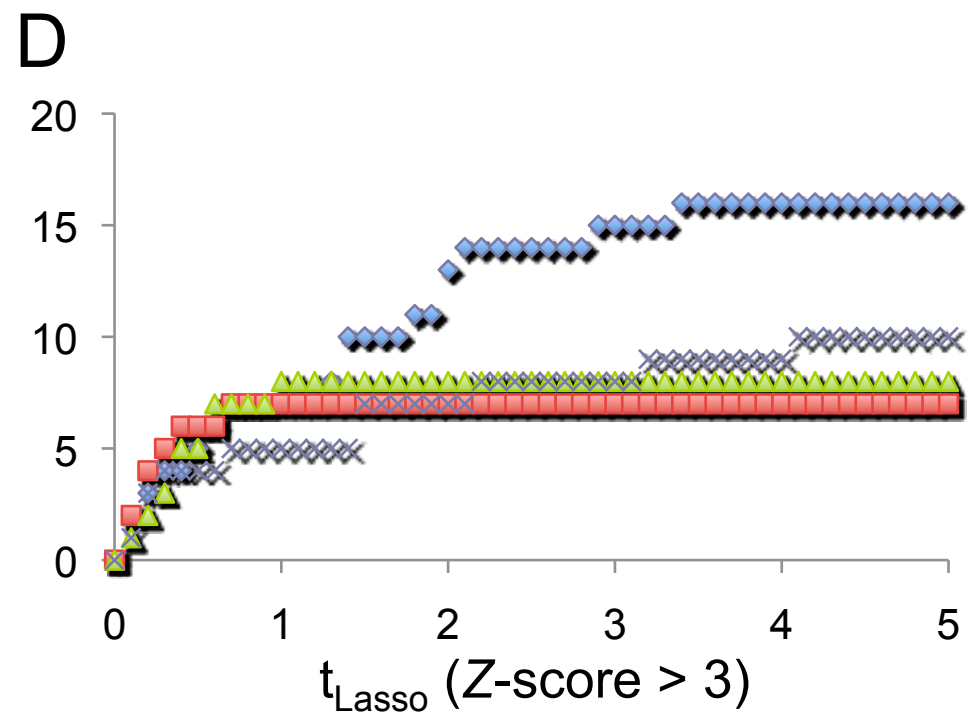

Supplement: Figure S3 — Phenotype prediction (number of fruits per plant, fruit harvested, average fruit weight and pH) by using the genotype described in the 50 RILs in which transcript levels were measured. Pearson coefficient correlation (A,C) between the predicted and measured phenotypic profile and number of genes (B,D) selected by LASSO method as predictors for different thresholds of the fitting parameter (tLASSO). Note that we used two different z-score levels (z = 2, (A,B); and z = 3 (C,D)) to included genes as possible predictors to be selected by LASSO. The dashed line plotted in (A,B) shows the parameter, tLASSO, and the level of z-score used to constructed the relationship between phenotype and metabolome. (PDF) [file pcbi.1002528.s007.pdf]

**A**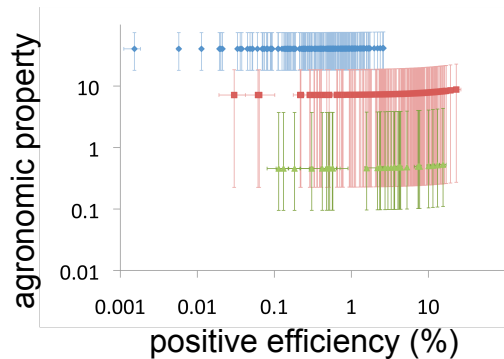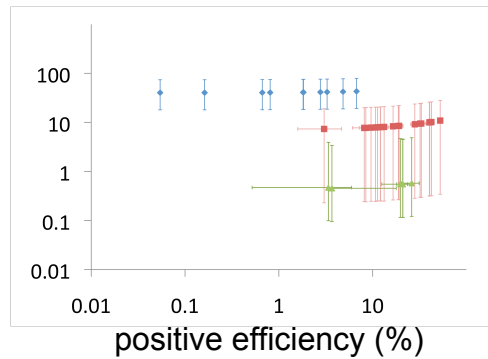**B**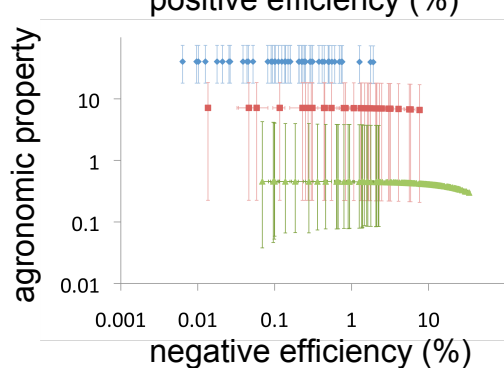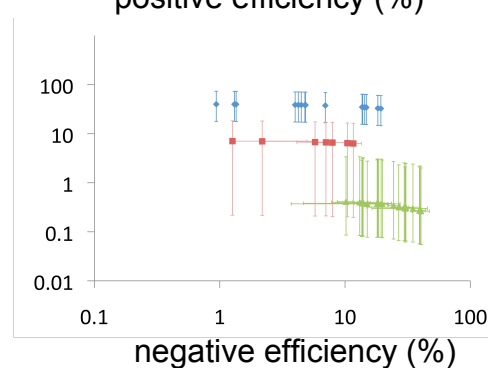**C**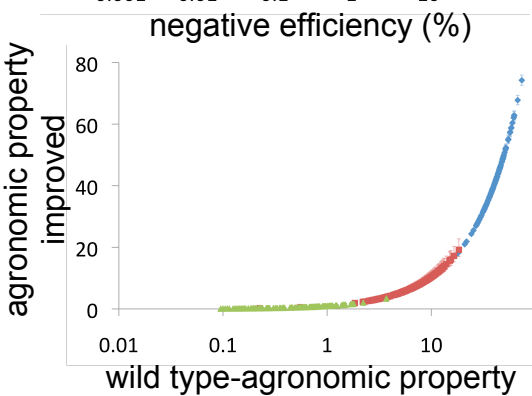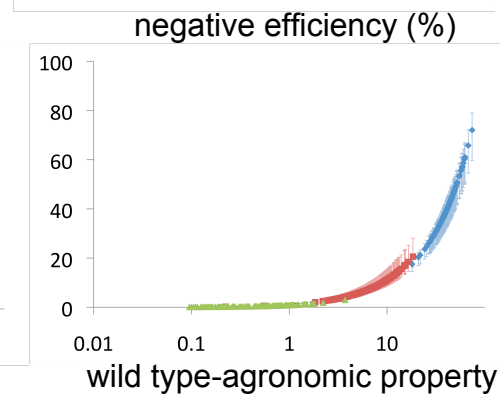**D**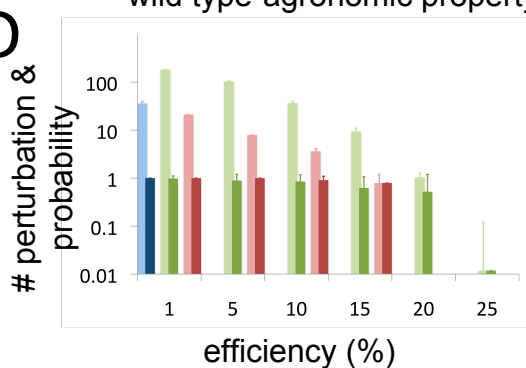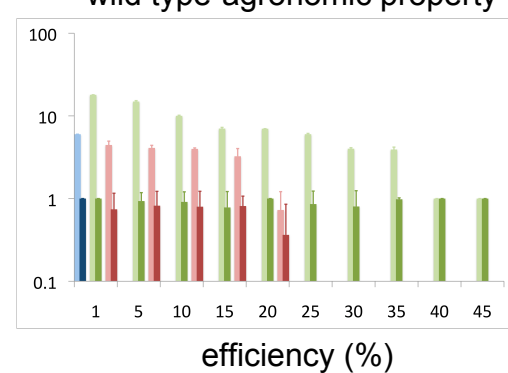

Supplement: Figure S4 — Exhaustive exploration and statistical significance of the landscape of single desired agronomic properties of tomato fruit (vitamin C, blue; fructose and glucose, red; and citric and malic acids, green) perturbing its effective TRN locally. (A) Agronomic properties improved by perturbing a single gene as function of efficiency reached by that transcriptional perturbation with respect to the wild-type scenario; notice that only perturbations with positive mean efficiencies are plotted. Both agronomic properties and efficiencies of a single perturbation are average variables tested on the 169 RILs and error bars represent their minimum and maximum values in both axis. (B) Dependence between agronomic properties in the wild-type genome and the average of the agronomic properties resulting of all single perturbations in the wild-type TRN for each RIL; vertical error bars represent the best and worst optimized re-engineered TRN for a given RIL. (C–D) Average number of single gene perturbations that overcome an efficiency threshold in the 169 RILS (light bars; error bars represent standard deviation for the 169 RILs) and average probability of selecting the same gene-perturbation commonly in a set of RILs (dark bars; error bars show standard deviation for all genes of the TRN). Left and right columns represent perturbations in terms of single gene knockout or overexpression, respectively. (PDF) [file pcbi.1002528.s008.pdf]

A

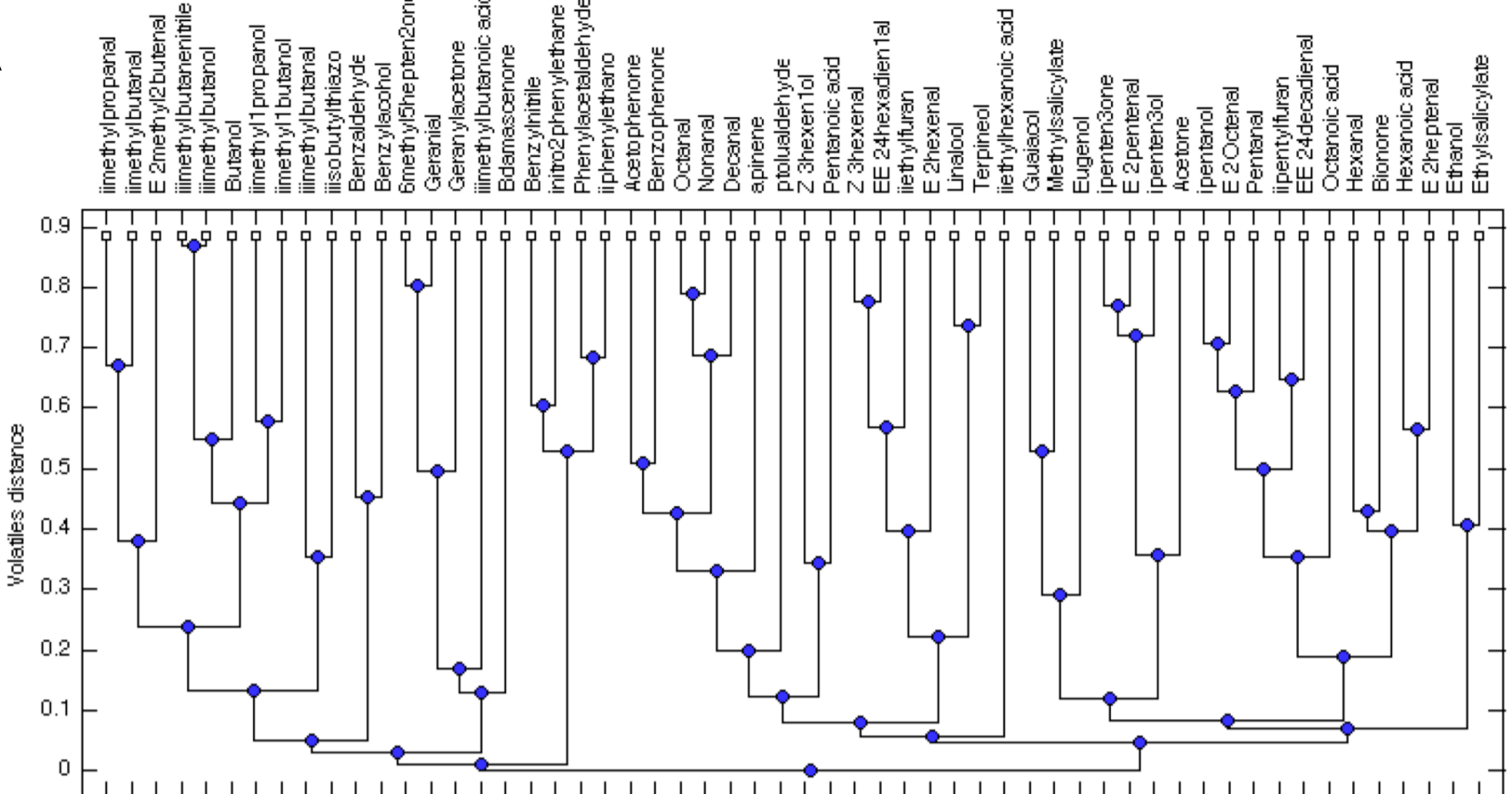

B

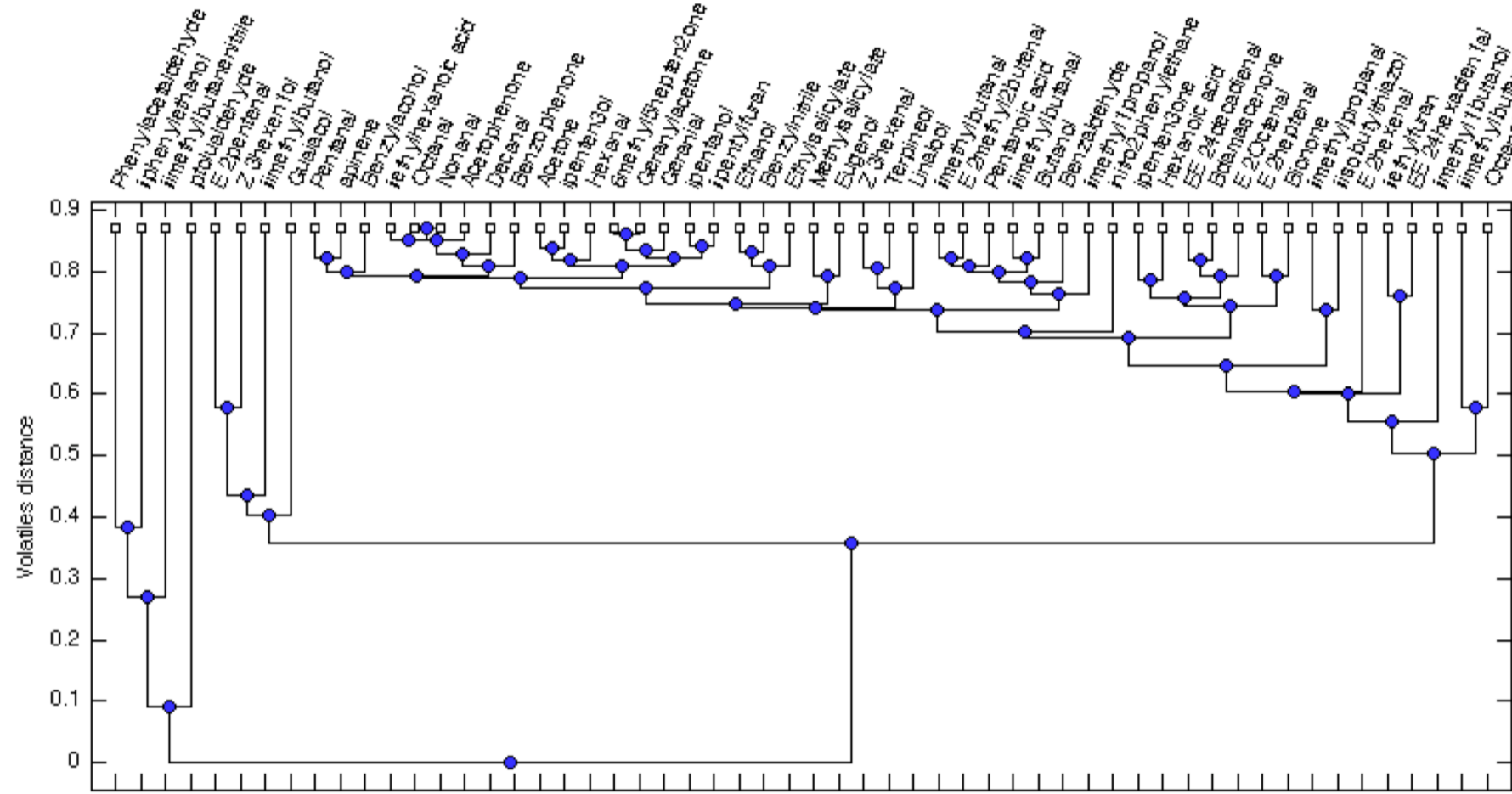

C

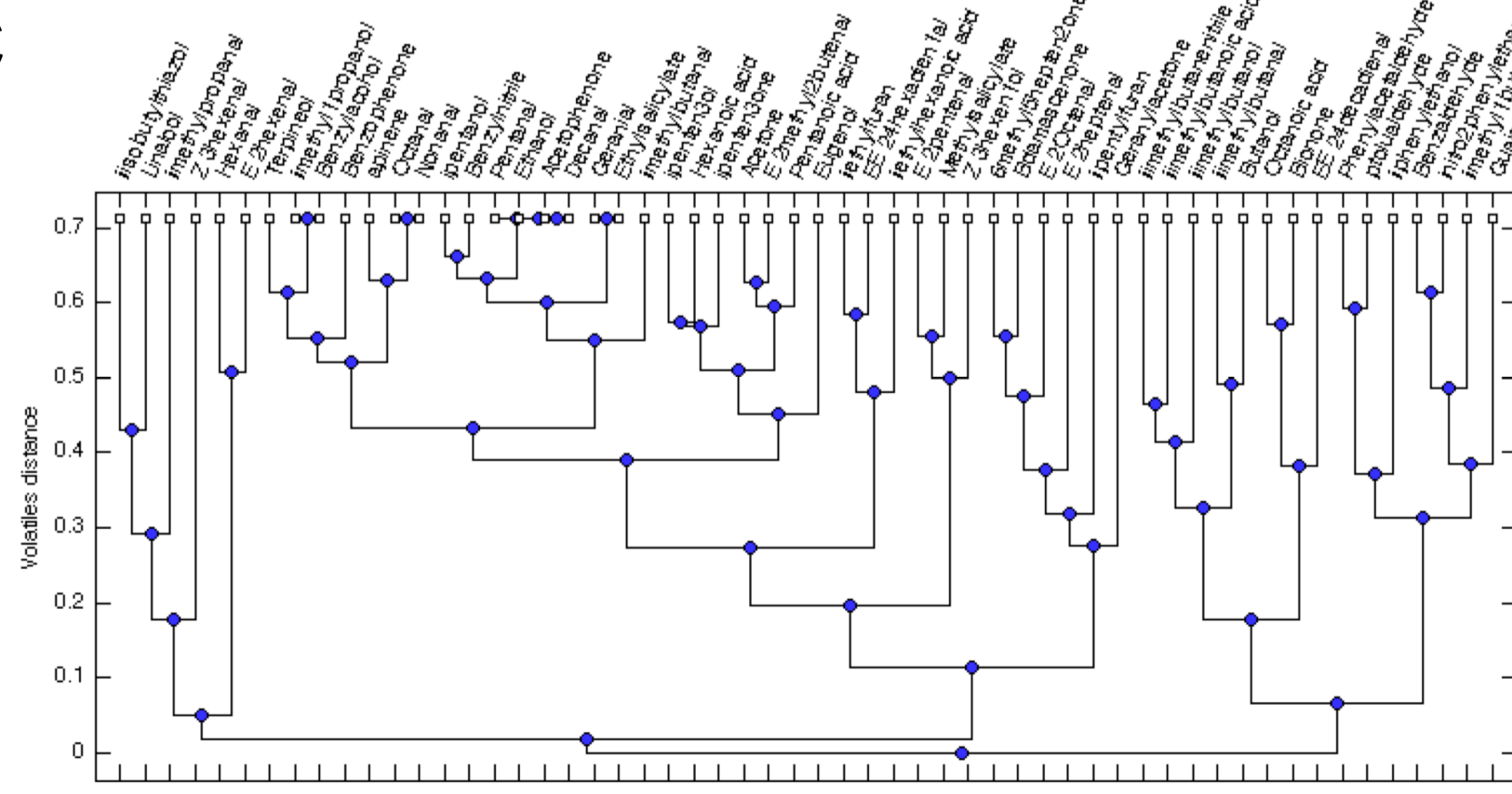

Supplement: Figure S5 — (A) Dendogram of the volatile compound correlations observed experimentally. (B, C) Dendograms inferred by the model defining the distance between volatile compound as the number of common genetic perturbations predicted to optimize the levels of each volatile compound. (PDF) [file pcbi.1002528.s009.pdf]

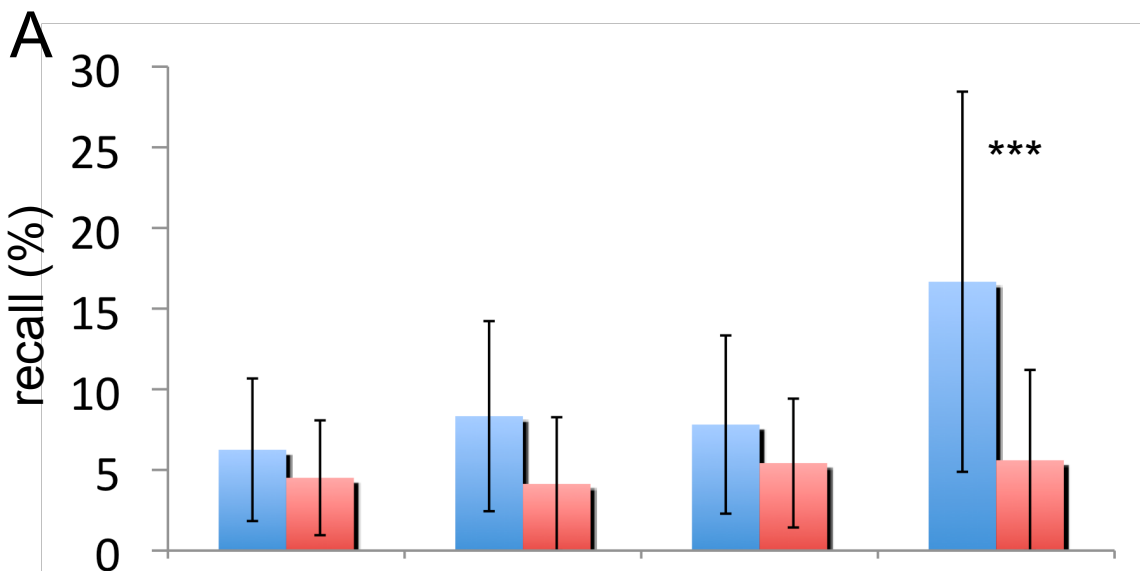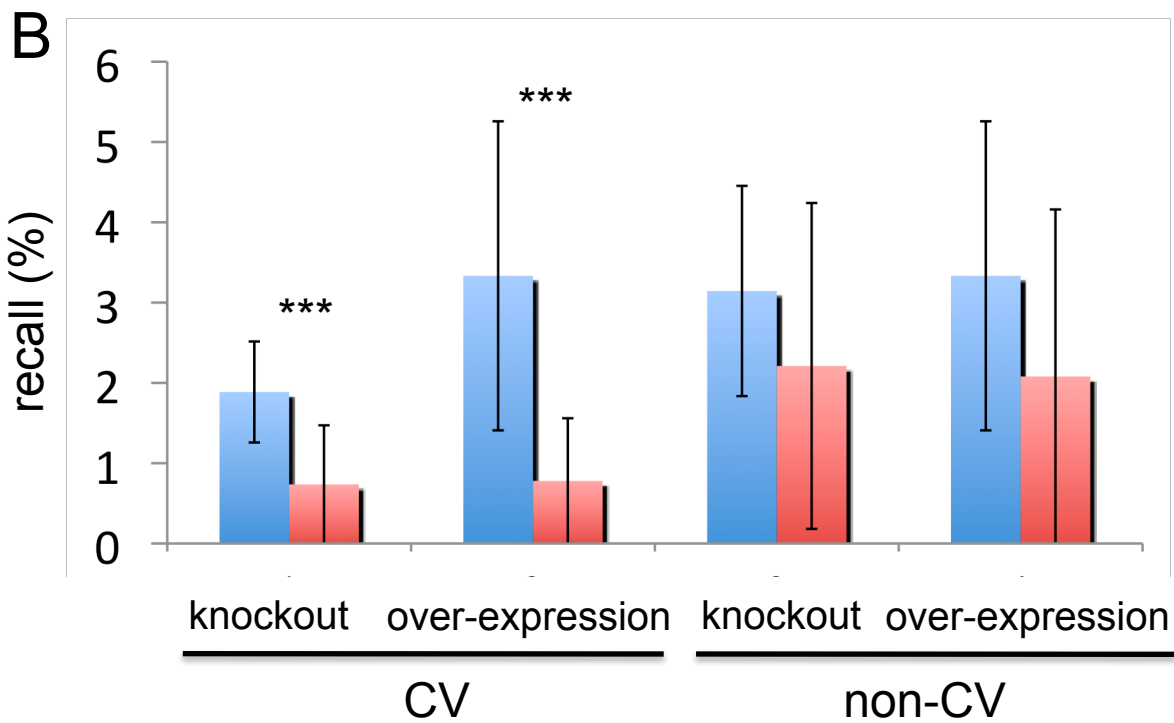

Supplement: Figure S6 — Percentage of altered genes (via gene knockout or over-expression; blue bars) proposed by the model to minimize the levels of volatile compounds (linalool (A) or, 1-nitro-2-phenylethane, 2-isobutylthiazole and benzylnitrile (B)) that were found significantly over-/under-expressed in the transcriptome of two ILs characterized experimentally with extremely low levels of those volatile compounds. The cut-off of the coefficient of variation between replicates was 75%. The Mann-Withney's U-test significance using random selection of gene perturbations (red bars) is shown (***statistically significant). Error bars represent the standard deviations of scores obtained from three ILs. 16.7% of the over-expressed genes proposed by the model to minimize the level of linalool were significantly recovered in gene expression (Figure S4A). In addition, 1.89% and 3.33% of genes candidates to be knockout or over-expressed (Figure S4B), respectively, also were identified significantly altered in the gene expression of the IL in which the three volatile compounds were found in minimum amount indicating this part of the transcriptome is relevant and associated to this volatile sub-phenotype among the other differential traits in these ILs. (PDF) [file pcbi.1002528.s010.pdf]

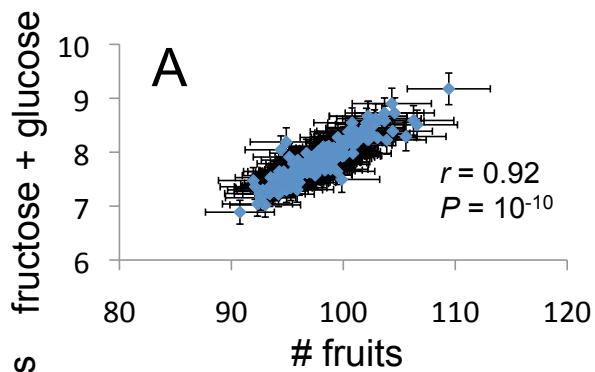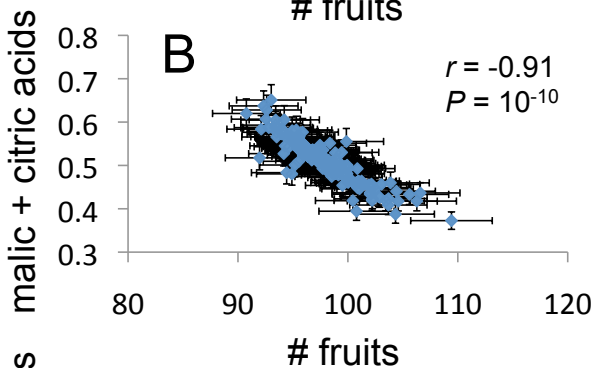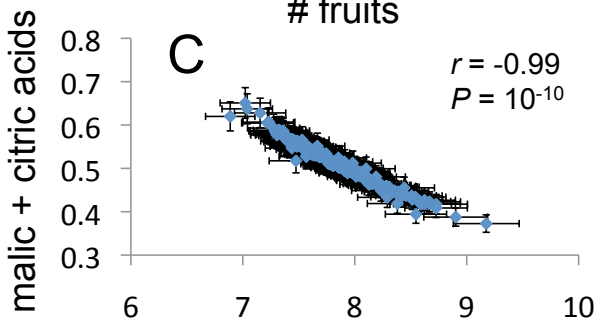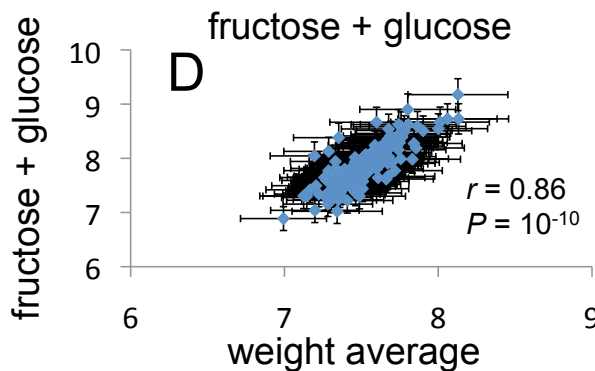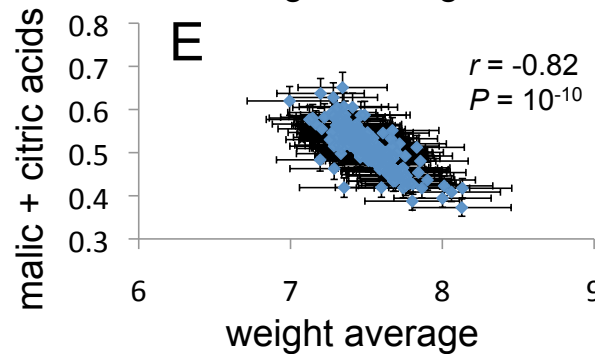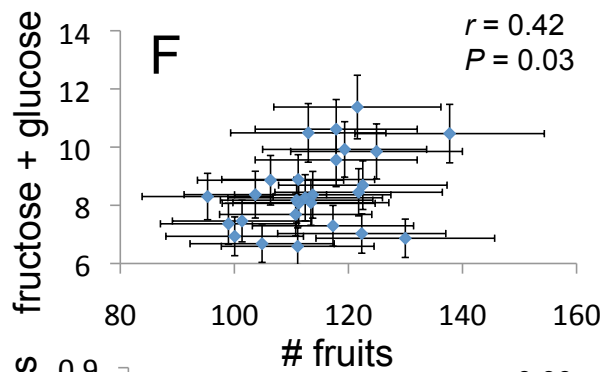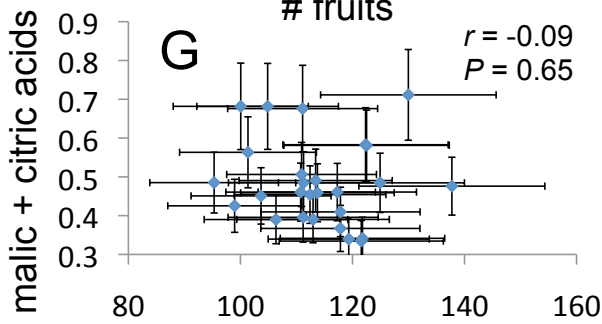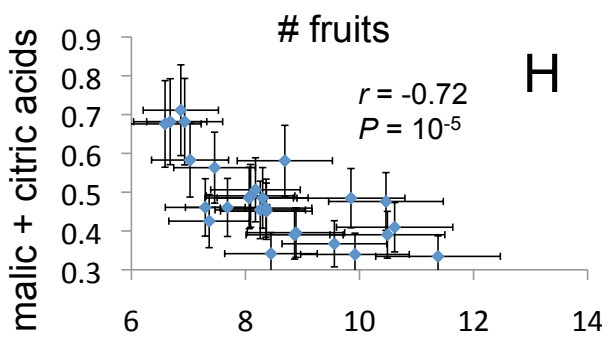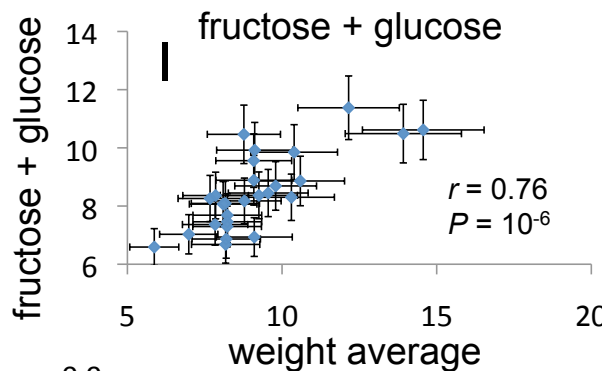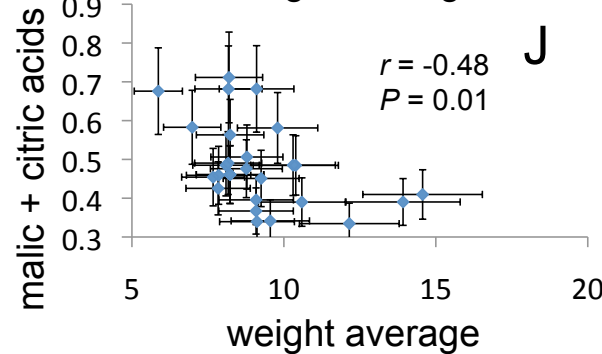

Supplement: Figure S7 — Correlations observed between agronomic variables and metabolites of different fruit genotypes generated by simulating all possible single gene knockout (A–E) or over-expression (F–J) in the wild-type genome model of the tomato fruit. Standard deviations of all metabolites or agronomic variables show the diversity generated by implementing each genetic perturbation in the 169 RILs. Note that we only plotted re-engineered genomes whose transcriptome predicted showed errors lower than 1% (241 d.f. and 25 d.f. for knockout and over-expressed genes, respectively). (PDF) [file pcbi.1002528.s011.pdf]
