# Supplementary material for: Fine-Tuning Tomato Agronomic Properties by Computational Genome Redesign
Source: PLoS Comput Biol. 2012 Jun 7;8(6):e1002528. doi: 10.1371/journal.pcbi.1002528 (PMC3369923; doi:10.1371/journal.pcbi.1002528)
Supplement: Table S1 — The top 5 single-gene knockouts and over-expressions that maximize the agronomic properties of the tomato fruit based on improve only one objective. (PDF) [file pcbi.1002528.s012.pdf]

Table S1. The top 5 single-gene knockouts and over-expressions that maximize the agronomic properties of the tomato fruit based on improve only one objective.

Gene	Gene Annotation	Efficiency (%)†	RIL	Probability‡
**Vitamin C**				
LE4I20	Serine/threonine protein kinase, putative	2.61	15	1
LE8C21	Tubulin-specific chaperone C-related	2.55	15	1
LE3E12	ABO1, transcription elongation regulator	2.37	15	1
LE29C10	Adenylate kinase 1, putative	2.19	15	1
LE28L05	Longevity assurance factor, putative	1.98	15	1
LE20K20	Cation efflux protein/zinc transporter, putative	6.74	15	1
LE16D22	Superoxide dismutase [Cu-Zn] 2	4.84	15	1
LE12J12	Chloroplast methionine sulfoxide reductase B1 precursor	3.27	15	1
LE12P18	Alcohol dehydrogenase, putative	2.77	15	1
LE4K23	Triosephosphate isomerase, chloroplastic	1.83	15	1
**Fructose and glucose**				
LE13F23	Chloroplast phosphate transporter precursor	23.66	86	0.01
LE8C21	Tubulin-specific chaperone C-related	19.88	86	0.05
LE26N09	6-Phosphogluconolactonase-like protein	17.45	86	1
LE2C24	ATAB2; RNA binding	17.41	86	0.99
LE32B05	YABBY2-like transcription factor YAB2	16.56	86	0.98
LE15D07	Polynucleotide kinase- 3'-phosphatase, putative	52.65	86	1
LE8A19	Putative glycerophosphoryl diester phosphodiesterase family protein	42.42	86	1
LE27C02	Phytoene dehydrogenase, chloroplastic/chromoplastic	40.59	86	1
LE14B20	Clathrin adaptor complexes medium subunit	40.56	86	0.92

	family protein			
LE12J12	Chloroplast methionine sulfoxide reductase B1 precursor	52.49	86	0.01
**Malic and citric acids**				
LE33H21	Chloroplast inner membrane import protein Tic22, putative	16.88	13	0.79
LE20D08	Cyclic nucleotide-gated calmodulin-binding ion channel	13.99	13	1
LE25M02	Auxin response factor 8	12.05	13	1
LE25A03	Ribosomal protein S27-like protein	9.63	13	0.63
LE18N13	Not found	8.03	13	1
LE16L04	Ureide permease, putative	31.00	13	0.02
LE1K08	Not found	25.90	13	1
LE23D07	Transcription factor-related	23.38	13	0.79
LE13M10	Ribosomal protein L30e	21.37	13	0.02
LE14J14	SRFR1, protein complex scaffold	5.80	13	0.04

Notice that the first five genes is the top 5 of single-gene knockouts (light rows) and the following five is the top 5 in over-expression (dark rows).

† Efficiencies were selected in the RIL where the perturbation maximizes the fitness.

‡ Probability of selecting the given perturbation across the set of RILs at the maximum level of efficiencies.
