# Supplementary material for: Fine-Tuning Tomato Agronomic Properties by Computational Genome Redesign
Source: PLoS Comput Biol. 2012 Jun 7;8(6):e1002528. doi: 10.1371/journal.pcbi.1002528 (PMC3369923; doi:10.1371/journal.pcbi.1002528)
Supplement: Table S2 — The top 5 single-gene knockouts and over-expressions that minimize the agronomic properties of the tomato fruit based on improve only one objective. (PDF) [file pcbi.1002528.s013.pdf]

**Table S2. The top 5 single-gene knockouts and over-expressions that minimize the agronomic properties of the tomato fruit based on improve only one objective.**

| Gene | Gene Annotation | Efficiency (%)† | RIL |
|---|---|---|---|
| **Vitamin C** | | | |
| *LE33G09* | Not found | -1.91 | 28 |
| *LE33H21* | Chloroplast inner membrane import protein Tic22, putative | -1.77 | 28 |
| *LE9K02* | Not found | -1.27 | 28 |
| *LE14J12* | 40S Ribosomal protein S3a-like | -0.75 | 28 |
| *LE17I11* | Potential GTPase activation protein | -0.73 | 28 |
| *LE14J14* | SRFR1, protein complex scaffold | -19.31 | 28 |
| *LE14B20* | Clathrin adaptor complexes medium subunit family protein | -18.28 | 28 |
| *LE27C02* | Phytoene dehydrogenase, chloroplastic/chromoplastic | -14.78 | 28 |
| *LE15D07* | Polynucleotide kinase- 3'-phosphatase, putative | -14.35 | 28 |
| *LE3H15* | Non-cell-autonomous protein pathway1, plasmodesmal receptor | -13.84 | 28 |
| **Fructose and glucose** | | | |
| *LE25A03* | Ribosomal protein S27-like protein | -7.66 | 24 |
| *LE33H21* | Chloroplast inner membrane import protein Tic22, putative | -5.33 | 24 |
| *LE20D08* | Cyclic nucleotide-gated calmodulin-binding ion channel | -5.19 | 24 |
| *LE25M02* | Auxin response factor 8 | -3.63 | 24 |
| *LE9K02* | Histone H3.2 | -2.98 | 24 |
| *LE13M10* | Ribosomal protein L30e | -12.73 | 24 |
| *LE1K08* | Not found | -10.46 | 24 |
| *LE16L04* | Ureide permease, putative | -9.27 | 24 |
| *LE23D07* | Transcription factor-related | -6.34 | 24 |
| *LE1B05* | Integral membrane Yip1 family protein | -6.83 | 24 |

| | | | |
|---|---|---|---|
| **Malic and citric acids** | | | |
| *LE13F23* | Chloroplast phosphate transporter precursor | -34.10 | 159 |
| *LE8C21* | Tubulin-specific chaperone C-related | -31.50 | 159 |
| *LE18G02* | Heat shock protein, putative | -30.91 | 159 |
| *LE2C24* | ATAB2; RNA binding | -27.77 | 159 |
| *LE26N09* | 6-Phosphogluconolactonase-like protein | -26.09 | 159 |
| *LE15D07* | Polynucleotide kinase- 3'-phosphatase, putative | -42.18 | 159 |
| *LE12J12* | Chloroplast methionine sulfoxide reductase B1 precursor | -40.03 | 159 |
| *LE29L04* | ER33 protein | -40.45 | 159 |
| *LE20K20* | Cation efflux protein/ zinc transporter, putative | -34.99 | 159 |
| *LE27I24* | Katanin P80 subunit, putative | -31.69 | 159 |

Notice that the first five genes is the top 5 of single-gene knockouts (light rows) and the following five is the top 5 in over-expression (dark rows).

† Efficiencies were selected in the RIL where the perturbation maximizes the fitness.
