# Supplementary material for: Fine-Tuning Tomato Agronomic Properties by Computational Genome Redesign
Source: PLoS Comput Biol. 2012 Jun 7;8(6):e1002528. doi: 10.1371/journal.pcbi.1002528 (PMC3369923; doi:10.1371/journal.pcbi.1002528)
Supplement: Text S1 — Genome design based on single perturbations to fine-tuning phenotypes with biotechnological interests. Model validation: fine-tuning tomato phenotype of two experimental inbred lines by computational genome design. Prediction of phenotypic correlations in re-engineered tomato fruits. (PDF) [file pcbi.1002528.s014.pdf]

Supplementary Text

Fine-tuning tomato agronomic properties by computational genome redesign

Javier Carrera, Asun Fernández del Carmen, Rafael Fernández-Muñoz, Jose Luis Rambla, Clara Pons, Alfonso Jaramillo, Santiago F. Elena, Antonio Granell

Contents

Supporting Text

Genome design based on single perturbations to fine-tuning phenotypes with biotechnological interests

Model validation: fine-tuning tomato phenotype of two experimental inbred lines by computational genome design

Prediction of phenotypic correlations in re-engineered tomato fruits

Supporting references

Supplementary Data

Supplementary Figures

Glossary

Supporting Text

Genome design based on single perturbations to fine-tuning phenotypes with biotechnological interests

We wonder whether we could be able to tune an agronomic property towards a defined value, as it is desired for some biotechnological applications. As examples, **Figure S4** (A, B) shows the range of values around the wild-type value of vitamin C, sugars (glucose and fructose) and malic and citric acids that could be obtained by means of single-gene transcriptional perturbations. Furthermore, when agronomic properties as well as associated efficiencies for single-perturbations were computed for the 169 RILs the results presented a highly significant linear correlation ($R^2 > 0.99$; $p < 0.001$) between both variables (agronomic values *vs* efficiencies).

We ranked the genes to be knockout/over-expressed in the TRN according to their mean efficiency across all lineages (**Data S2**). **Tables S1** and S**2** show the top 5 genes that when altered would either maximize or minimize the corresponding evaluated fitness. Vitamin C content in fruits could be increased a 6.74% or decreased a 19.31% in all lineages by over-expressing gene *LE20K20* or *LE14J14*, respectively. Glucose and fructose content could be highly increased reaching improvement ratios of 52.65% by over-expressing *LE15D07*; it could also be decreased a 12.73% by over-expressing *LE13M10*. In the case of the main acids contributing to tart flavor in tomato: malic and citric acids, maximal ratios could be increased to a 31.00% (i.e., *LE16L04*) or decreased a 42.18% by using gene over-expression. **Figure S4C** shows the strong linear dependence between agronomic properties calculated for wild-type TRN and the average values of the agronomic properties resulting from all single perturbations when applied to the TRN for each RIL (linear regression slope in the

range 0.99 - 1.12, $R^2 > 0.99$, 167 d.f., $p < 0.001$). Also in this case we observed that genetic perturbations increased the number of lineages with maximum agronomic properties in the wild-type TRN, this confirming the high level of robustness of our model when we selected the lineages to implement re-designed TRN. **Figure S4D** (right panel) shows that only a few gene knockouts were able to improve fruit acceptability with a high probability in all lineages whereas tens of different gene knockouts would have a high probability of being selected in different lineages designed to increase fruit quality (similar case for overcoming the trade-off between quality and production). As can be seen in **Figure S4D** (left panel) re-engineering the TRN by gene over-expression provided better improvements in the agronomic properties. Over-expression also seems to provide a higher density of perturbations as it is indicated by the results across the RILs.

Model validation: fine-tuning tomato phenotype of two experimental inbred lines by computational genome design

The development of technologies to synthesize new genomes and to introduce them into hosts with inactivated wild-type chromosomes is opening the door to new horizons in synthetic genomics[1]. Several initial reports have been focused on the chemical synthesis, assembly, and expression of viral genomes[2-3]. Engineering more complex organisms poses additional challenges including the definition of the gene networks underlying traits of interest[4-7]. It is therefore of outmost importance to harness the ability of using computational design to predict and optimize a synthetic genome before attempting its synthesis (**Figure S1**)[8-9].

Our hypothesis has been that high-throughput technologies combined with rigorous and biologically rooted modeling will allow us to predict the effect of simple genetic perturbations on the dynamic behavior of cellular genetic and metabolic networks.

Transcriptomics, metabolomics and phenomics need to be properly integrated to formulate a model that can be used to predict quantitatively how transcriptional changes can result in desired phenotypic responses of the tomato fruit in its red ripe stage. The final goal would be the accurate prediction of quantitative information of agronomic or biotechnological interest and the possibility for re-engineering cellular circuits. To reach this goal, we have succeeded in the integration of experimental and computational approaches to construct an effective regulatory network model that covers a large extent of the transcriptome and metabolome of tomato, specially those metabolites related to fruit quality.

Hence, to give further support to our model we designed experimentally two ILs whose transcriptome and metabolome were measured. Those two ILs were characterized by minimizing the levels of linalool and a set of three volatile compounds (1-nitro-2-phenylethane, 2-isobutylthiazole and benzylnitrile), respectively. At this point, could RILs-based global model suggest consistent genetic perturbations in the set of RILs to obtain the same phenotypic response observed in the ILs? To address this question, we imposed the phenotypic constraints as design specifications in our model to obtain a set of genetic perturbations that mimicked the phenotype measured. Two different sets of candidate genes to be under- or over-expressed were proposed by the model to optimize both ILs (**Data S2**). We recovered a significant overlapping in the set of genes proposed by the model with respect to the genes differentially expressed in those lines (**Figure S6**), indicating that these overlapping genes can be contributing to the low levels of the selected volatiles in those lines (the other differentially expressed genes are probably related to other traits segregating in this population). These genes are the targets of future studies in tomato.

Prediction of phenotypic correlations in re-engineered tomato fruits

Our reverse-engineering approach provides the first quantitative global model that integrates high-throughput data related to transcriptional regulatory and metabolic processes in order to describe important aspects of the phenotype of tomato fruits with high predictability. There are previous reports in the literature of quantitative models in simpler biological systems[10] but there is a lack of similar work in complex multi-cellular organisms. Previous reports on integration of data sets in complex systems including tomato have revealed the network of interactions using different approaches and visualization/cartographic methods[11] but never reaching or proposing a quantitative model.

Systems biology studies aimed at integrating omics results in tomato have been focused in understanding the fruit ripening process and normally have used wild-type and ripening mutants[12-13]. Other studies have targeted the metabolite/transcript fruit network and the effect of environment or early fruit development in the final fruit phenotype[14]. Similar integrative analysis of complex data have been also conducted in tomato in relation to targeted fruit biosynthetic pathways resulting in correlation and visualization maps based on wild-type and a limited number of transgenics affected in genes of those pathways (e.g., vitamin C[15]). Our approach here used natural genetic perturbations introduced by the different introgressions of the tomato's close relative *S. pimpinellifolium* into a series of tomato RILs that result in large phenotype variability in fruits at the red ripe stage. Previous reports have also used tomato variability in the form of ILs to address at a genomic level the basis for metabolism and yield associated traits by mapping and co-localization of markers associated to these traits[11, 16]. Our analysis here was not intended to just reveal de correlations between metabolites and transcripts but to

produce a working model for engineering tomato fruit quality. Furthermore it allowed us to infer biotechnological strategies from the design of a library of synthetic genomes that incorporated single gene knockout or over-expression in the wild-type genome. Using these *in silico* re-engineered genomes we were able to predict significant correlations that were not easily detected in the initial dataset such as negative correlation between the number of fruits or the weight average of each fruit and its levels of acids; surprisingly, re-engineered genomes with high levels of sugars showed a strong positive correlation with the number of fruits or their weight average (**Figure S7**).

Supporting references

1. Gibson, D. G., Benders, G. A., Andrews-Pfannkoch, C., Denisova, E. A., Baden-Tillson, H., Zaveri, J., Stockwell, T. B., Brownley, A., Thomas, D. W., Algire, M. A., Merryman, C., Young, L., Noskov, V. N., Glass, J. I., Venter, J. C., Hutchison III, C. A., Smith, H. O. Complete chemical synthesis, assembly and cloning of a *Mycoplasma genitalium* genome, *Science* **319**, 1215–1220 (2008).

2. Blight, K. J., Kolykhalov, A. A., Rice C. M. Efficient Initiation of HCV RNA Replication in Cell Culture. *Science* **290**, 1972 (2000).

3. Cello, J., Paul, A. V., Wimmer, E. Chemical Synthesis of Poliovirus cDNA: Generation of Infectious Virus in the Absence of Natural Template. *Science* **297**, 1016 (2002).

4. Smith, H. O., Hutchison III, C. A., Pfannkochm C., Venter, J. C. Generating a synthetic genome by whole genome assembly: fX174 bacteriophage from synthetic oligonucleotides. *Proc. Natl. Acad. Sci. U. S. A.* **100** (2003).

5. Kodumal, S. J., Patel, K. G., Reid, R., Menzella, H. G., Welch, M., Santi, D. V. Total synthesis of long DNA sequences: Synthesis of a contiguous 32-kb polyketide synthase gene cluster. *Proc. Natl. Acad. Sci. U. S. A.* **101**, 15573 (2004).

6. Lartigue, C., Glass, J. I., Alperovich, N., Pieper, R., Parmar, P. P., Hutchison III, C. A., Smith, H. O., Venter, J. C. Genome transplantation in bacteria: changing one species to another. *Science* **317**, 632–638 (2007).

7. Dymond, J. S. et al., *Nature*, doi:10.1038/nature10403 (2011).

8. Carrera, J., Rodrigo G., Jaramillo, A. Towards the automated engineering of a synthetic genome. *Mol. Biosyst.* **5**, 733-743 (2009).

9. Barrett C. L., Kim, T. Y., Kim, H. U., Palsson, B. Ø., Lee, S. Y. Systems biology as a foundation for genome-scale synthetic biology. *Curr. Op. Biotech.* **17**, 488-492 (2006).

10. Carrera, J., Rodrigo, G., Jaramillo, A. Model-based redesign of global transcription regulation. *Nucleic Acids Res.* **37**, e38 (2009).

11. Schauer, N., Semel, Y., Roessner, U., Gur, A., Balbo, I., Carrari, F., Pleban, T., Perez-Melis, A., Bruedigam, C., Kopka, J., Willmitzer, L., Zamir, D., Fernie, A. R. Comprehensive metabolic profiling and phenotyping of interspecific introgression lines for tomato improvement. *Nat. Biotech.* **24**, 447-54 (2006).

12. Osorio, S., Alba, R., Damasceno, C. M., Lopez-Casado, G., Lohse, M., Zanor, M. I., Tohge, T., Usadel, B., Rose, J. K., Fei, Z., Giovannoni, J. J., Fernie, A. R. Systems biology of tomato fruit development: combined transcript, protein, and metabolite analysis of tomato transcription factor (nor, rin) and ethylene receptor (Nr) mutants reveals novel regulatory interactions. *Plant Physiol.* **157**, 405-25 (2011).

13. Rohrmann, J., Tohge, T., Alba, R., Osorio, S., Caldana, C., McQuinn, R., Arvidsson, S., van der Merwe, M. J., Riaño-Pachón, D. M., Mueller-Roeber, B., Fei, Z., Nesi, A. N., Giovannoni, J. J., Fernie, A. R. Combined transcription factor profiling, microarray analysis and metabolite profiling reveals the transcriptional control of metabolic shifts occurring during tomato fruit development. *Plant J.* doi: 10.1111/j.1365-313X.2011.04750.x (2011).

14. Mounet, F., Moing, A., Garcia, V., Petit, J., Maucourt, M., Deborde, C., Bernillon, S., Le Gall, G., Colquhoun, I., Defernez, M., Giraudel, J. L., Rolin, D., Rothan, C., Lemaire-Chamley, M. Gene and metabolite regulatory network analysis of early developing fruit tissues highlights new candidate genes for the control of tomato fruit composition and development. *Plant Physiol.* **149**, 1505-28 (2009).

15. Garcia, V., Stevens, R., Gil L., Gilbert, L., Gest, N., Petit, J., Faurobert, M., Maucourt, M., Deborde, C., Moing, A., Poessel, J. L., Jacob, D., Bouchet, J. P., Giraudel, J. L., Gouble, B., Page, D., Alhagdow, M., Massot, C., Gautier, H., Lemaire-Chamley, M., de Daruvar, A., Rolin, D., Usadel, B., Lahaye, M., Causse, M., Baldet, P., Rothan, C. An integrative genomics approach for deciphering the complex interactions between ascorbate metabolism and fruit growth and composition in tomato. *C R Biol.* **332**, 1007-21 (2009).

16. Kamenetzky, L., Asís, R., Bassi, S., de Godoy, F., Bermúdez, L., Fernie, A. R., Van Sluys, M. A., Vrebalov, J., Giovannoni, J. J., Rossi, M., Carrari, F. Genomic analysis of wild tomato introgressions determining metabolism- and yield-associated traits. *Plant Physiol.* **152**, 1772-86 (2010).

Supplementary data

Dataset S1 (xls file). Transcriptional, metabolic and phenotypic models of tomato fruit.

Dataset S2 (xls file). Single knockout and over-expressed genes to improve desired agronomic properties (acceptability, quality and quality *vs* production of tomato fruits; four volatile compounds; vitamin C, and different types of sugars and acids) and functional categorization of genes that induced high degree of improvement in those agronomic properties; notice that functional enrichment of all genes involved in the TRN was included. Gene ontology enrichment analyses were performed using the TFGD tool [TFGD]. It is also showed the functional categories significantly represented among those genes that were selected to describe the TRN of tomato fruit. A total of 19 cellular processes and 45 biological components were represented. Among these, genes related to cellular metabolic processes were the most abundant ($p < 0.0001$), what makes sense since they were selected to predict cellular metabolism; whereas genes related to response to nutrient stimulus were present but the least common ($p < 0.1$).

Dataset S3 (xls file). Multiple combinations of knockout and over-expressed gene sets to improve desired agronomic properties (acceptability, quality and quality *vs* production of tomato fruits).

Dataset S4 (xls file). Experimental evidences of each gene perturbation proposed by the model to optimize the different scoring function used.

Supplementary figures

Figure S1. Synthetic biology of tomato fruit *vs* computer science.

Figure S2. From data to global models to redesign using an approach based on synthetic biology.

Figure S3. Phenotype prediction (number of fruits per plant, fruit harvested, average fruit weight and pH) by using the genotype described in the 50 RILs in which transcript levels were measured. Pearson coefficient correlation (A,C) between the predicted and measured phenotypic profile and number of genes (B,D) selected by LASSO method as predictors for different thresholds of the fitting parameter (t_{LASSO}). Note that we used two different z-score levels (z = 2, (A,B); and z = 3 (C,D)) to included genes as possible predictors to be selected by LASSO. The dashed line plotted in (A,B) shows the parameter, t_{LASSO}, and the level of z-score used to constructed the relationship between phenotype and metabolome.

Figure S4. Exhaustive exploration and statistical significance of the landscape of single desired agronomic properties of tomato fruit (vitamin C, blue; fructose and glucose, red; and citric and malic acids, green) perturbing its effective TRN locally. (A) Agronomic properties improved by perturbing a single gene as function of efficiency reached by that transcriptional perturbation with respect to the wild-type scenario; notice that only perturbations with positive mean efficiencies are plotted. Both agronomic properties and efficiencies of a single perturbation are average variables tested on the 169 RILs and error bars represent their minimum and maximum values in both axis. (B) Dependence between agronomic properties in the wild-type genome and the average of the agronomic properties resulting of all single perturbations in the wild-type TRN for each RIL; vertical error bars represent the

best and worst optimized re-engineered TRN for a given RIL. (C-D) Average number of single gene perturbations that overcome an efficiency threshold in the 169 RILS (light bars; error bars represent standard deviation for the 169 RILs) and average probability of selecting the same gene-perturbation commonly in a set of RILs (dark bars; error bars show standard deviation for all genes of the TRN). Left and right columns represent perturbations in terms of single gene knockout or over-expression, respectively.

Figure S5. (A) Dendogram of the volatile compound correlations observed experimentally. (B, C) Dendograms inferred by the model defining the distance between volatile compound as the number of common genetic perturbations predicted to optimize the levels of each volatile compound.

Figure S6. Percentage of altered genes (via gene knockout or over-expression; blue bars) proposed by the model to minimize the levels of volatile compounds (linalool (E) or, 1-nitro-2-phenylethane, 2-isobutylthiazole and benzylnitrile (F)) that were found significantly over-/under-expressed in the transcriptome of two ILs characterized experimentally with extremely low levels of those volatile compounds. The cut-off of the coefficient of variation between replicates was 75%. The Mann-Withney's *U*-test significance using random selection of gene perturbations (red bars) is shown (***statistically significant). Error bars represent the standard deviations of scores obtained from three ILs. 16.7% of the over-expressed genes proposed by the model to minimize the level of linalool were significantly recovered in gene expression (Figure S4A). In addition, 1.89% and 3.33% of genes candidates to be knockout or over-expressed (Figure S4B), respectively, also were identified significantly altered in the gene expression of the IL in which the three volatile compounds were found in minimum amount indicating this part of the

transcriptome is relevant and associated to this volatile sub-phenotype among the other differential traits in these ILs.

Figure S7. Correlations observed between agronomic variables and metabolites of different fruit genotypes generated by simulating all possible single gene knockout (A-E) or over-expression (F-J) in the wild-type genome model of the tomato fruit. Standard deviations of all metabolites or agronomic variables show the diversity generated by implementing each genetic perturbation in the 169 RILs. Note that we only plotted re-engineered genomes whose transcriptome predicted showed errors lower than 1% (241 d.f. and 25 d.f. for knockout and over-expressed genes, respectively).

Glossary

A recombinant inbred line (**RIL**) population is a collection of basically homozygous lines that derive from a cross between two different genotypes after a series of consecutive steps. RILs contain different sets of interdispersed genome fragments from each of the parental lines, in our case the cultivated variety of *S. lycopersicum* and a wild accession of *S. pimpinellifolium*, but always in a 50/50 percent proportion.

Introgression lines (**ILs**) are basically a series of homozygous lines that contain a small and defined fragment of one genome (in our case from the wild species *S. pennellii*) in a background of the genome of the same or related species (in our case the cultivated species, *S. lycopersicum*). They derive from an initial cross between the two species and are constructed by genetic-marker selection within populations obtained after several backcrosses to one of the parental lines (in this case the cultivar M82) and a few steps to achieve homozygosis.
